# Performance and Tolerance of a Protocol for Idiopathic Chronic Greasy Seborrhea in 18 Dogs Using a Shampoo and Mousse Containing Plant Extracts

**DOI:** 10.3390/vetsci10020095

**Published:** 2023-01-28

**Authors:** Jevgenija Kondratjeva, Jessie Brun, Nicolas Amalric, Fabien Moog, Daniel Combarros, Charline Pressanti, Claudine Zemirline, Nadège Maubert, Elodie Ollivier, Marina Gatellet, Marie Christine Cadiergues

**Affiliations:** 1 Small Animal Clinic, Université de Toulouse, ENVT, 31076 Toulouse, France; 2 QIMA Life Sciences, 31670 Labège, France; 3 INFINITy, Université de Toulouse, Inserm, CNRS, UPS, 31059 Toulouse, France; 4 Ceva Santé Animale, 53950 Louverné, France; 5 Ceva Santé Animale, 33500 Louverné, France

**Keywords:** canine, skin, seborrhea, shampoo, mousse, Ophytrium, Seboliance

## Abstract

**Simple Summary:**

Greasy skin and excessive skin scaling accompany numerous dermatoses. Rapid and sustainable improvement of skin appearance and application practicability are important features to enhance the dog’s appearance and encourage owner compliance. The study aimed to evaluate the tolerance and performance of one shampoo and subsequent mousses applications containing plant extracts in dogs with greasy skin. Six dogs were washed with plain water on day (D)0 and served as negative controls. Twelve dogs were shampooed on D0 and received eight mousse applications at 48–72 h intervals from D2 to D18. Skin was evaluated on D0, D0 + 4 h, D7, D14 and D24. At baseline, there were no significant differences observed between groups. In the control group, skin appearance and lipid contents remained stable throughout the study. Skin lesions and malodour were significantly reduced in the test group from D7 with no side effects. Hydration and hair lipids levels decreased in the test group at D0 + 4 h and returned to baseline from D14 and D7 on, respectively. In conclusion, one shampoo and subsequent mousse applications rapidly and safely improved coat and skin appearance in dogs with greasy skin and dandruff without affecting water and hair lipid contents.

**Abstract:**

The study aimed to evaluate the tolerance, performance and effect on hair lipids and skin hydration of a protocol combining applications of one shampoo and subsequent mousses containing plant extracts (Ophytrium and Seboliance) in dogs with an undiagnosed chronic greasy keratinisation disorder. Six dogs were washed with plain water on day (D)0. Twelve dogs were shampooed on D0 and received eight mousse applications at 48–72 h intervals from D2 to D18. Clinical score (CS), Natural Moisturizing Factors (NMF) and hair lipids (HL) were evaluated on D0, D0 + 4 h, D7, D14 and D24. At baseline, no significant differences were observed in CS, NMF and HL between groups. In the control group, CS and HL remained stable throughout the study while a slight decrease in NMF was observed at D0 + 4 h. CS was significantly reduced in the test group between D0 and D7 (−53%) which reached 91% at D24 (*p* < 0.05), with no side effects. NMF levels decreased in the test group at D0 + 4 h (−73%, *p* < 0.0001) and returned to baseline from D14. In conclusion, one shampoo and subsequent mousse applications rapidly and safely improved coat quality in dogs with an undiagnosed keratinisation disorder without affecting NMF and HL contents over the study period.

## 1. Introduction

In mammals, sebaceous glands and keratinocytes produce a protective lipid layer that covers the skin [1]. The majority of epidermal surface lipids are of sebaceous origin while the lipids produced by the epidermis act as an insignificant fraction of the total extractable surface lipid [2]. Depending on the condition of the skin, different lipids are found in variable quantities and proportions [3]. In dogs, there are much fewer data. A recent study has shown the very large diversity of lipids found on the surface of the *stratum corneum* in the West Highland white terrier breed [4], without being able to determine whether their origin was mainly glandular as in humans.

Keratinisation disorders are clinical conditions characterized by an alteration of the balance between cell death and renewal of keratinocytes, possible excessive greasiness and/or increased scale formation on the skin; it can be secondary to inflammation or be a primary skin disease. In dogs, secondary keratinisation disorders predominate and have a wide range of causes, including inflammatory diseases (ectoparasites, bacterial pyoderma, *Malassezia* overgrowth, atopic dermatitis), endocrinopathies, nutritional imbalances or environmental factors. The greasiness and scaling of the skin in dogs with keratinisation disorders have been reported to be associated with alteration of the lipid profile of sebaceous glands and the *stratum corneum* [1,5]. The primary cause of a given keratinisation disorder is not always straightforward and easy to control. Topical therapy is important in the management. It can be used as a sole or as an adjunctive therapy for these disorders, often with the aim of minimising the need for systemic therapy [6]. Shampoos are often considered as the most effective of the topical therapy options [6]. Nevertheless, this process is time consuming, requires commitment, efforts and in many cases, specific space and equipment. Over the past decade, manufacturers have sought easy-to-use formulations.

Further, various plant-derived ingredients have been identified and are suggested to minimize the severity of skin scaling [7,8]. *Ophiopogon japonicus* (common names: dwarf lilyturf, Mondo grass, fountainplant, monkeygrass) is a perennial evergreen plant of the Liliaceae family and is mainly distributed in southern China, Japan, Vietnam and India [9]. The main components of the roots of *O. japonicus* include steroidal saponins, homoisoflavonoids and polysaccharides [10,11]. *O. japonicus* is known to be an effective anti-inflammatory herbal medicine in many Asian countries [11]. Ophytrium has been assessed in vitro in reconstructed epidermis models [12,13]. It limited the adhesion and biofilm formation of *Staphylococcus pseudintermedius* at the surface of the reconstructed canine epidermis [13], and was shown in vitro to have beneficial effects on the mechanical and immunological skin barriers [12].

*Punica granatum* (common name: Pomegranate) belongs to the Punicaceae family and is widely cultivated throughout the Middle East and Caucasus region, north and tropical Africa, Iran, the Indian subcontinent, Central Asia, the drier parts of Southeast Asia and the Mediterranean Basin [14]. Several parts of this plant contain numerous phytochemical compounds (flavonoids, polyphenols, tannins, organic acids, etc) [15]. Recent studies reported innate properties of some phytoextracts from *P. granatum*, including antioxidant, antiseborrheic, anti-inflammatory, anticarcinogenic, antiviral and antifungal properties [15,16,17]. *P. granatum* extracts have antimicrobial properties in vitro, as demonstrated by the inhibitory effect of an extract on the bacterial growth of *S. aureus* and *Escherichia coli* [18], and multidrug-resistant *S. epidermidis* strains [19]. Another extract from *P. granatum* showed an anti-inflammatory potential in vitro [20]. In a study of anti-acne properties of pomegranate in rat, antibacterial, anti-lipase, anti-keratinocyte proliferation and anti-inflammatory actions were reported [21]. An ear cleanser containing pomegranate extract combined with prednisolone showed antifungal activity in the auditory canals of dogs with otitis externa [22].

The aim of the present study was to evaluate the performance and tolerance of two formulations of keratomodulating products—shampoo and mousse (DOUXO^®^ S3 SEB, Ceva Santé Animale, Libourne, France)—containing extracts from the roots of *O. japonicus* (Ophytrium, Ceva Santé Animale proprietary) and the peel of *P. granatum* (Seboliance, Ceva Santé Animale proprietary) in seborrheic dogs.

## 2. Materials and Methods

### 2.1. Ethics

The protocol was approved by the Sciences et Santé Animales (SSA—Ecole Nationale Vétérinaire Toulouse) n°115 Ethics Committee (approval no. SSA_2019_006). The dogs’ owners gave written consent prior to the study.

### 2.2. Animals

Eighteen adult hound dogs of three different breeds (Bruno du Jura, Bleu de Gascogne and Griffon), with scaly and greasy skin of several-month duration but otherwise clinically healthy were included in the study. They lived in the same geographic area in groups of one to five dogs in semi-open kennels. The inclusion criteria were animals in good general health as confirmed by a general physical examination by a licensed veterinarian and the absence of clinical signs such as gastrointestinal signs, lethargy, obvious lameness, respiratory signs or cutaneous signs other than those consistent with a keratinisation disorder (greasy skin/haircoat, malodour, scaling, erythema and alopecia). Exclusion criteria were pregnancy and lactation, parasitic infestation, allergic skin conditions, skin infection, endocrine diseases and any disease or treatment which could influence the evaluation of the skin during the study. The animals should not have received any treatment for fungal or bacterial skin infections or oral glucocorticoids in the four weeks preceding the study, nor any application of topical antiseptics or anti-inflammatory treatment over the two weeks preceding the study nor of any topical product (shampoo, spray, mousse) in the week preceding inclusion.

Hematology and full biochemistry profiles were all unremarkable. Ectoparasites (fleas, *Cheyletiella*, lice, scabies and demodicosis) had been ruled out appropriately by coat combing, skin scraping and hair plucking and coproscopy (feacal flotation) was negative for all dogs. Allergic skin conditions (atopic dermatitis and flea allergy dermatitis) were excluded based on the history, the absence of significant pruritus and the absence of lesions such as erythema, alopecia or lichenification on the typically affected body regions [23]. Dogs were on monthly antiparasitic prevention (Credelio comprimés à croquer pour chiens, Elanco France, Sèvres, France).

The dogs were permanently housed in semi open kennels on clay courts. Water was provided *ad libitum*. All the dogs had been receiving the same dry, balanced diet (Pasqui Energie, agriPasquier, Les Cerqueux, France) for the past six months and their diet was not modified during the study. The environmental and housing conditions and level of care of all the dogs remained unchanged throughout the study.

### 2.3. Design of the Study

This was a prospective, randomized, controlled 24-day study. Each dog was allocated a number from 1 to 18 for the duration of the study. Before beginning the study, the 18 dogs were randomly (simple randomization, Research Randomizer (Version 4.0) [24]) assigned to group A (negative control group; six dogs) or group B (DOUXO^®^ S3 SEB Shampoo and Mousse, Ceva Santé Animale, Libourne, France group; 12 dogs). The two groups were housed in separate kennels and never came into direct or indirect contact with dogs in the other group during the course of the study.

The six dogs in group A were hand washed with plain water (temperature of approx. 15 °C) on day (D) 0 for 10 min and received no further intervention. In group B, the 12 dogs were shampooed on D0 with the test shampoo (1 pump dose/2 kg for short-haired dogs and 1 pump dose/kg in dense and/or long-haired dogs). The shampoo was applied on a pre-wet haircoat, massaged and left on for ten minutes before rinsing. All dogs were left to air dry. Dogs subsequently received an application of DOUXO^®^ S3 SEB Mousse, Ceva Santé Animale, Libourne, France on D2, 4, 7, 9, 11, 14, 16 and 18 (1 pump dose/2 kg for short-haired dogs and 1 pump dose /kg in dense and/or long-haired dogs). All applications were carried out by trained staff who were not involved in the clinical evaluation. The owner was instructed to monitor any adverse event and to report to the investigator in due course. The study was conducted during the closed hunting season.

### 2.4. Clinical Assessment

After a general clinical examination, the dogs were dermatologically evaluated on D0, D7, D14 and D24. A scoring index, adapted from Viaud et al. [25] was used which allowed attribution of a clinical score (CS). Briefly, the following parameters were assessed on a 0–3 scale: malodour, scaling, greasiness, haircoat quality and extent of the affected area (Table 1). The maximum possible CS was 15. The intensity of pruritus was evaluated by the owner using a visual analogue scale [26] on the same days.

### 2.5. Skin Surface Cytology

To evaluate an eventual proliferation of bacteria and/or yeasts on the skin surface, surface cytologies were performed. Acetate tape preparations were performed on D0, 7, 14 and 24 by repeatedly pressing a 1 × 1 cm area of a strip of clear acetate tape (Scotch^®^ Crystal; 3 M; Cergy-Pontoise, France) against the most affected site of the skin for five seconds before removing it. Collected samples were analysed microscopically after staining (RAL555^®^; RAL Diagnostics; Site Montesquieu-Martillac, France). Ten high power fields (HPF) were examined at ×1000 magnification and cytological findings were scored semi-quantitatively using a 0–4 scale as described by Bouassiba et al. [27].

### 2.6. Natural Moisturising Factor (NMF) Content

In order to evaluate a possible variation in skin hydration during the study, the content of NMFs was measured, as NMFs are hydrophilic markers of *stratum corneum* [28]. Skin surface samples were taken for NMFs analysis on D0, D0 + 4 h, D7, D14 and D24 by rubbing a 5-cm line on the skin with two swabs (Dryswab^®^ MWE, Medical Wire & Equipment, Wiltshire, UK) previously soaked in an aqueous non-ionic surfactant solution (QIMA, Labège, France—proprietary method), using a standardised, previously validated, sampling technique (unpublished data). The swab heads were then removed, placed in dry Eppendorf tubes (Safe-Lock Tubes 1.5 mL, Eppendorf AG, Hamburg, Germany) and frozen at −20 °C until analysis.

NMFs of interest (urocanic acid (cis/trans-UCA), pyrrolidone carboxylic acid (PCA) and serine were extracted using an aqueous solution, then analysed by a LC/MS system (UltiMate 3000 liquid chromatography system (ThermoScientific, Sunnyvale, CA, USA) coupled with a MSQ Plus Mass detector (ThermoScientific, Sunnyvale, CA, USA). Results are expressed in µg per sample.

### 2.7. Hair Surface Lipids

To assess the impact of products applications on the amounts of lipids in the coat, hair surface lipids were quantified. Hair samples were collected from the dorsum of each dog by shaving hair shafts with a clipper (Aesculap Isis clipper GT421, B Braun Medical; Saint Cloud, France) on D0, D0 + 4 h, D7, D14 and D24. An area of approximately 480 mm² was clipped on the dorsum of each dog on each sampling occasion. Samples were conserved in plastic vials (Greiner Bio-one CELLSTAR^®^, Greiner Bio-One, Kremsmünster, Austria) at −20 °C until analysis.

Before analysis, 100 mg of hair fibres were cut and washed in a mixture of water, sodium dodecylsulphate and hexane. Total lipids were extracted using the method of Bligh and Dyer [29]. Mass fraction results are expressed in µg of total lipids per mg of hair fibres.

### 2.8. Tolerance

After the shampoo was applied, tolerance was evaluated on the following in-house scale: very bad (withdrawal movement, defence movement, or pruritic behaviours with vocalization), bad (withdrawal movement, defence or pruritic behaviours), medium (pruritic behaviours after application), good (rare pruritic behaviours after application) or excellent (no pruritic behaviours after application). Adverse events occurring in between visits were recorded.

### 2.9. Investigator and Owner Satisfaction

At the end of the study, the following items were evaluated on a 0–4 scale (0- very bad, 1—bad, 2—average, 3—good, 4—excellent): overall assessment of tolerance by the investigator; overall assessment of tolerance by the owner; overall assessment of performance by the investigator and overall assessment of performance by the owner.

### 2.10. Statistical Methods

The Shapiro–Wilk normality test was used to test data for normality distribution. When the distribution of the data was normal, Bartlett’s test was used to verify homogeneity of variance and data collected on the different sampling days were compared using the parametric ANOVA 1 test. For data that showed statistically significant differences over time, Bonferroni’s multiple comparison test was computed to compare individual time points within the group. In the absence of a normal distribution at all-time points, non-parametric Mann–Whitney tests were used. Statistical analyses were performed using XLSTAT software (Microsoft^®^, version Base 2019.4.1.63305) and a two-sided *p*-value < 0.05 was considered statistically significant.

## 3. Results

### 3.1. Animal Population

Group A (negative control group) included five male dogs and one female (five Griffons and one Bruno du Jura). The median age was 4 years (range 2–5) and median weight 27.3 kg (range 24–33). Group B (DOUXO^®^ S3 SEB group) comprised 10 male dogs and 2 females (six Griffons, three Bruno du Jura and three Bleu de Gascogne), median age 5 years (range 1–8), median weight 27.0 kg (range 20–29). The differences in breed, sex, age or weight between the two groups were not statistically significant. Details are given in Table 2.

### 3.2. Clinical Score

**Figure 1:**
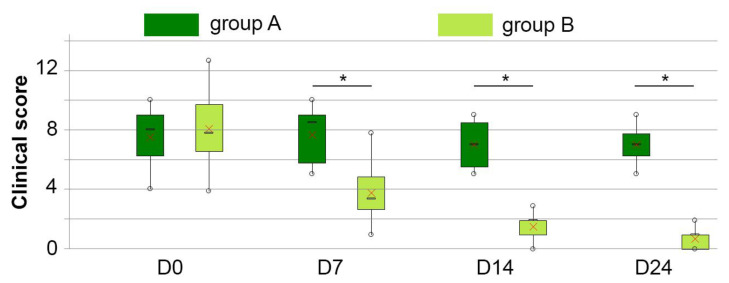
Kinetics of clinical scores in dogs with keratinization disorders that were washed in plain water (group A—dark green boxes) or received DOUXO^®^ S3 SEB shampoo and mousse (Ceva Santé Animale, France) (group B—light green boxes) at different time points during the study. Interpretation of box and whisker plots: lower and upper box boundaries 25th and 75th percentiles, respectively, line inside box median, lower and upper whiskers minimum and maximum scores, respectively. X represents the mean. * represents significant differences at *p* = 0.05, compared to baseline values.

The baseline clinical scores were similar in the two groups (median 8, min 4, max 10 and 13, group A and B, respectively) and in group A, the clinical scores remained steady throughout the study (median between 7 and 8.5, min 4 or 5 and max 9 or 10, *p* > 0.05). Conversely, a statistically significant reduction in CS was observed in group B on D7 compared to on D0 (median 3.5, min 1, max 8) (*p* = 0.002), reaching the lowest score on D24 with 91% of reduction (median 1, min 0, max 2) (*p* < 0.001)—Table 3 and Figure 1.

Changes were observed in all five parameters of the CS (Table 4), the most dramatic changes were in the malodour score (−93% on D7 and −100% on D14).

### 3.3. Pruritus

On D0, pruritus was very mild and similar in the two groups (median 0.5/10, min 0.4 and 0.2, max 1.1 and 1.0, group A and B, respectively). Its severity remained very low (<0.4) throughout the study in the two groups (Table 3).

### 3.4. Skin Surface Cytology

No evidence of microbial elements was found on D0 or at any time point throughout the study.

### 3.5. NMF Content

**Figure 2:**
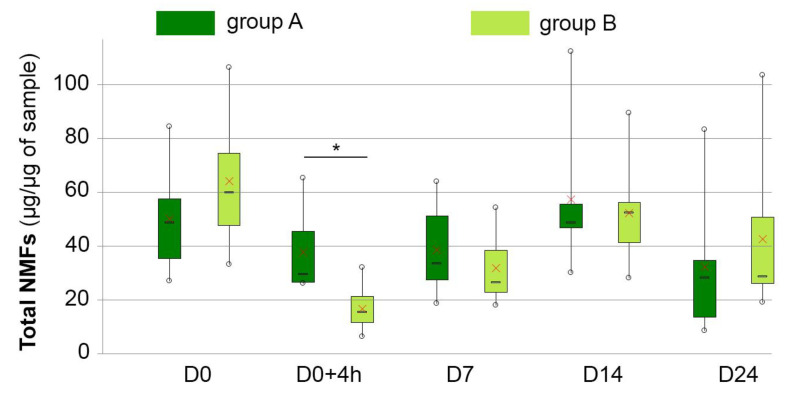
Kinetics of natural moisturizing factors (NMFs) in dogs with keratinization disorders that were washed in plain water (group A—dark green boxes) or received DOUXO^®^ S3 SEB shampoo and mousse (Ceva Santé Animale, France) (group B—light green boxes) at different time points during the study. Interpretation of the box and whisker plots: lower and upper box boundaries 25th and 75th percentiles, respectively, line inside box median, lower and upper whiskers minimum and maximum scores, respectively. X represents the mean. * represents significant differences at *p* = 0.05, compared to baseline values.

At baseline, NMF contents did not significantly differ between the two groups. In the control group, they decreased slightly at D0 + 4 h, then returned to normal at D14, then decreased again between D14 and D24. NMF levels decreased in the test group at D0 + 4 h (−73%, *p* < 0.0001), returned to baseline from D14 on and were then slightly higher than in the control group at D24. Details are given in Table 3 and Figure 2.

### 3.6. Hair Lipid Analysis

**Figure 3:**
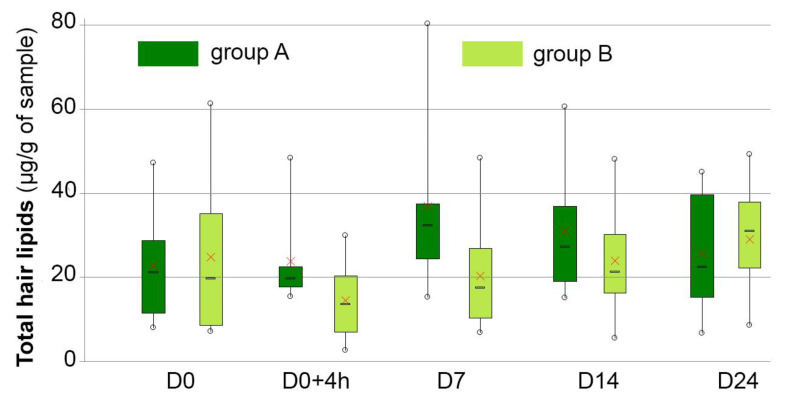
Kinetics of hair lipids in dogs with keratinization disorders that were washed in plain water (group A—dark green boxes) or received DOUXO^®^ S3 SEB shampoo and mousse (Ceva Santé Animale, France) (group B—light green boxes) at different time points during the study. Interpretation of the box and whisker plots: lower and upper box boundaries 25th and 75th percentiles, respectively, line inside box median, lower and upper whiskers minimum and maximum scores, respectively. X represents the mean.

At baseline, lipid contents did not differ significantly between the two groups. In the control group, lipid contents remained stable throughout the study. Lipid levels decreased in the test group at D0 + 4 h (−50%, *p* = 0.14) and remained lower than those of the hair of control dogs at D7 and D14, but the difference was not significant (*p* = 0.08 and *p* = 0.494). At D14, the levels were back to baseline. Details are given in Table 3 and Figure 3.

### 3.7. Tolerance and Feedback from the Users

The shampoo was well tolerated. No adverse reaction was reported after shampoo and mousse applications. Tolerance and the cosmetic effect were scored 4/4 by both the owner and the investigator. Efficacy and practicability were scored 3/4 by both the owner and the investigator.

## 4. Discussion

In this study, the combination of one shampoo and subsequent mousse applications at 48–72 h intervals was a convenient protocol to quickly reduce greasiness, scaling, malodour and improve subjectively hair coat quality in dogs with an undiagnosed keratinisation disorder. Canine keratinization disorders may improve with regular appropriate shampoos [6], but today simpler and/or easier to implement protocols are desired, especially when long-term management is required. The application of a mousse is simple through massage, it does not require prior wetting of the skin, nor rinsing or drying afterwards, thus allowing longer direct skin contact and prolonged action [30]. In recent years, mousse formulations, containing either phytosphingosine and pseudofilaggrin [31], or plant extracts [32] have been used in the management of canine atopic dermatitis, in spontaneous cases, improving skin lesions and pruritus. In another study, the authors showed that a mousse containing phytosphingosine and pseudofilaggrin could decrease the skin pH and inflammation in an experimental model of impaired skin barrier [33]. Other mousse formulations have shown an in vitro residual effect against *Staphylococcus pseudintermedius* after application on the hair coat [34]. To the best of the authors’ knowledge, this is the first controlled study of the use of a mousse combined with a shampoo in the management of a keratinisation disorder in dog and confirms the results obtained with the same combination in a non-controlled study [35].

The clinical score improved from the first recheck (day 7), and continued to improve until the end of the study (day 24). Among all the criteria evaluated, the biggest change was in the malodour score. This criterion is considered by the owners as one of the main complaints in keratinisation disorders and one of the major reasons for using a shampoo [36]. Clinical signs were completely resolved in all but one dog in the tested group, achieving a total reduction of 95% on D24. In the present study, all the dogs of the tested group initially presented with scaling; the amount of scales decreased over the course of the study and disappeared in most of the treated dogs. The remaining dogs had a maximum score of 1 out of 3.

Plant compounds are very popular in human cosmetics, and some are also incorporated in formulations for animals [37]. However, few scientific data are available concerning their effectiveness [22,38,39,40,41,42]. In the present study, both the shampoo and the mousse formulations contained two ingredients of plant origin: Ophytrium is a specific extract from the tuberous roots of *O. japonicus* and Seboliance is a specific extract from the peel of *P. granatum.* The presence of scales in dermatological diseases is explained by abnormalities during the keratinisation and/or desquamation steps [5]. Shampooing probably helps as it mechanically removes some of the scales. However, in the present study, as only one shampooing was performed, it is hypothesized that anti-keratinocyte proliferation action of *P. granatum* extract played a role [21]. Nevertheless, to demonstrate this effect, a group of dogs receiving a shampoo similar to group B but lacking the Ophytrium and Seboliance would have been necessary.

Both formulations, shampoo and mousse, were well tolerated by all the treated dogs—no side effects were observed by the investigators during the study. The negative impact of harsher surfactants on the skin barrier in some antiseborrhoeic shampoos is sometimes a concern for prescribers and users [43]. This action leads to a notable loss of lipids, most often related to the surfactants contained in certain products [44,45]. In the present study, shampooing (D0) resulted in a moderate reduction in the amount of lipids, as expected after use of an anti-seborrheic shampoo. The amount of lipids found on the hair of tested dogs remained lower than on the hair of control dogs at D7 and D14; no rebound effect was observed. NMFs are the degradation products of the skin protein filaggrin, and consist primarily of amino acids or their derivatives such as PCA and UCA, together with lactic acid, urea, citrate and sugars [46]. NMFs regulate *stratum corneum* hydration, its pH and activity of enzymes involved in key homeostatic processes in the *stratum corneum* including lipid synthesis, maturation of keratinocyte cornified envelope and keratinocyte desquamation. In humans, reduced NMFs are associated with increased transepidermal water loss secondary to skin barrier dysfunction [47]. In the present study, after shampooing (D + 4 h), NMFs content helped (i) monitor the progression of the integrity of the skin barrier, which can sometimes be impaired by excessive or repeated cleaning actions, and (ii) assess the hydration performance of the products. Whether due to the shampoo (tested group) or to simply rinsing with plain water (control group), the quantities of NMFs decreased at D0 + 4 h, logically more markedly in the tested group than in the control group because of the surfactant action of the shampoo. The amounts of NMFs then gradually increased again, more markedly in the tested group than in the control group, to reach baseline levels from D14 on. It could suggest the mousse may have contributed to the restoration of the skin barrier by supplying hydrating and protecting ingredients, although only a separate study looking at shampoo only *vs* shampoo with mousse would be required to support adequately this statement. The results of the lipid and NMF assays confirm—at the biochemical level—the immediate and lasting tolerance to shampooing combined with multiple applications of mousse.

The present study has several limitations. As previously stated, it would have been interesting to have, in addition to the actual control group, a control group on which a similar shampoo without Ophytrium and Seboliance would have been tested. Similarly, a control mousse without the active ingredients would have been interesting to test. The number of dogs included in the study is relatively low. The severity of the skin disease based on the clinical score is fairly low, which may have affected the final scores obtained. Further studies, conducted on more severe cases are advisable. Finally, the study was single blinded (investigators were blinded but not the owner).

## 5. Conclusions

The combination of one shampooing and subsequent mousse, containing Ophytrium and Seboliance, applications every 48–72 h, was found as to be a convenient, significantly effective and safe protocol to rapidly reduce malodour, greasiness, scaling and improve subjectively hair coat quality in dogs with an undiagnosed keratinisation disorder, without dehydrating their skin. Clinical observations were supported by biochemical assays showing that this protocol, in addition to being effective, did not damage the skin and is likely suitable for a long-term use.

## Figures and Tables

**Table 1:** Grading scale used to evaluate skin lesions in dogs with keratinization disorders. The clinical score was obtained by adding the different scores and ranged between 0 and 15.

Parameter	0	1	2	3
malodour	absence	low	mild	high
scaling	absence	low	mild	high
greasiness	absence	low	mild	high
haircoat quality	normal	slightly altered	mildly altered	highly altered
extension (%body surface)	<20%	20–50%	50–75%	>75%

**Table 2:** Details of the distribution of the animals between the two groups of dogs with keratinization disorders that were washed in plain water (group A) or received DOUXO^®^ S3 SEB shampoo and mousse (Ceva Santé Animale, France) (group B).

Group	Breed	Gender	Age (Years) Median (Min–Max)	Weight (kg) Median (Min–Max)	Length of Haircoat
A	5 Griffon	4 male, 1 female	4 (4–5)	27 (24–33)	longhaired
	1 Bruno du Jura	1 male	2	33	shorthaired
B	6 Griffon	6 male	5.5 (1–7)	28 (25–28)	longhaired
	3 Bruno du Jura	3 male	6 (2–8)	28 (24–29)	shorthaired
	3 Bleu de Gascogne	1 male, 2 female	3 (3–6)	24 (20–25)	longhaired

**Table 3:** Results of the clinical and biochemical parameters at different time-points of the two groups of dogs with keratinization disorders that were washed in plain water (group A) or received DOUXO^®^ S3 SEB shampoo and mousse (Ceva Santé Animale, France) (group B).

Parameter	Group	D0	D0 + 4 h	D7	D14	D24
Clinical score (/15)	A	7.5 ± 2.3		7.7 ± 2.2	7.0 ± 1.8	7.0 ± 1.4
	B	8.3 ± 2.6		3.9 ± 2.0	1.6 ± 0.9	0.8 ± 0.8
Pruritus (/10)	A	0.6 ± 0.3		0.0 ± 0.0	0.2 ± 0.1	0.0 ± 0.0
	B	0.5 ± 0.2		0.0 ± 0.1	0.2 ± 0.1	0.0 ± 0.0
NMFs (µg/sample)	A	49.96 ± 20.72	37.72 ± 16.29	38.58 ± 17.76	57.20 ± 28.38	32.17 ± 27.49
	B	64.83 ± 25.36	17.43 ± 7.51	32.41 ± 12.15	53.02 ± 16.70	43.30 ± 28.11
Hair lipids (μg of total lipids per mg of hair fibers)	A	22.80 ± 14.86	23.85 ± 12.29	36.85 ± 22.93	31.05 ± 16.98	25.74 ± 15.88
	B	25.32 ± 18.27	15.25 ± 8.05	20.93 ± 11.94	24.43 ± 13.40	29.47 ± 12.52

**Table 4:** Details of the clinical scores (mean ± standard deviation) in dogs with keratinization disorders that were washed in plain water (group A) or received DOUXO^®^ S3 SEB shampoo and mousse (Ceva Santé Animale, France) (group B) at different time points during the study. The arrow indicates the trend for each parameter at each time-point, compared to D0.

Clinical Parameter	Group	D0	D7	D14	D24
malodour	A	1.2 ± 0.8	1.3 ± 0.5 →	1.8 ± 0.8 ↑	1.8 ± 0.4 ↑
	B	1.2 ± 0.7	0.1 ± 0.3 ↓	0.0 ± 0.0 ↓	0.0 ± 0.0 ↓
scaling	A	1.0 ± 0.9	1.5 ± 0.5 ↑	2.2 ± 0.4 ↑	1.2 ± 1.0 →
	B	1.6 ± 0.8	0.9 ± 1.0 ↓	0.8 ± 0.6 ↓	0.4 ± 0.5 ↓
greasiness	A	2.0 ± 0.6	1.7 ± 0.5 →	1.8 ± 0.4 →	1.7 ± 0.5 →
	B	1.7 ± 0.7	0.8 ± 0.6 ↓	0.8 ± 0.5 ↓	0.1 ± 0.3 ↓
haircoat quality	A	1.7 ± 0.5	1.7 ± 0.5 →	0.8 ± 0.8 ↓	1.3 ± 0.8 ↓
	B	2.0 ± 0.7	1.3 ± 0.5 ↓	0.0 ± 0.0 ↓	0.2 ± 0.4 ↓
Extension (%body surface)	A	1.7 ± 0.5	1.5 ± 0.8 →	0.3 ± 0.5 ↓	1.0 ± 0.6 ↓
	B	1.9 ± 0.7	0.8 ± 0.5 ↓	0.0 ± 0.0 ↓	0.1 ± 0.3 ↓

## Data Availability

The data presented in this study are available from the corresponding author upon request. The data are not publicly available due to the need to maintain patient confidentiality.
